# Ground-glass opacity heralding invasive lung adenocarcinoma with prodromal dermatomyositis: a case report

**DOI:** 10.1186/s13019-018-0705-x

**Published:** 2018-02-07

**Authors:** Andrew J. Beel, David S. Demos, Alfred Chung, Charles Liao, Natalie S. Lui

**Affiliations:** 1 Stanford University School of Medicine, Stanford, USA; 2 Division of Thoracic Surgery, Stanford University School of Medicine, Falk Building, 300 Pasteur Drive, Stanford, 94305 USA; 3 Division of Oncology, Stanford University School of Medicine / Stanford Cancer Institute, Stanford, USA; 4 Division of Hospital Medicine, Stanford University School of Medicine, Stanford, USA

**Keywords:** Dermatomyositis, Lung adenocarcinoma, Subsolid, Ground-glass opacity

## Abstract

**Background:**

Dermatomyositis, an inflammatory myopathy with cutaneous involvement, is associated with malignancy and often manifests paraneoplastically. While co-occurrence with small cell carcinoma is well attested, primary lung adenocarcinoma, which may present as focal ground-glass opacification on computed tomography of the thorax, is less frequently coincident.

**Case presentation:**

We report the case of a 72-year-old female patient with dermatomyositis — treated with a combination of prednisone, methotrexate, and intravenous immunoglobulin — and an indolent, subsolid, non-hypermetabolic pulmonary lesion, which was determined to be invasive primary lung adenocarcinoma. Supporting a paraneoplastic basis, immunosuppressive therapy was discontinued following tumor excision without relapse of signs or symptoms of dermatomyositis.

**Conclusions:**

While dermatomyositis prodromal to lung adenocarcinoma is not without precedent, association with an indolent, subsolid lesion has, to the best of our knowledge, not been reported. The case described herein illustrates the importance of maintaining a high index of suspicion for malignancy in the setting of dermatomyositis.

## Background

Idiopathic inflammatory myopathies are a group of diseases of uncertain but presumed autoimmune etiology characterized by weakness and inflammation of the proximal musculature, and include polymyositis (PM), dermatomyositis (DM), sporadic inclusion body myositis, and necrotizing autoimmune myopathy. The best characterized among them, PM and DM, effect insidious dysfunction of the proximal musculature in a symmetric fashion, the progression of which may produce such complications as dysphagia and respiratory failure. Despite their similarities, DM distinguishes itself from PM through a plethora of cutaneous manifestations, including “Gottron’s papules” on the dorsal aspects of the phalangeal joints, the periorbital heliotrope eruption, and rashes distributed characteristically over the back (“shawl sign”), chest (“V sign”), or thighs (“holster sign”) [1]. Since the diagnostic codification of DM and PM by Bohan and Peter [2], many proposals for systematizing the diagnosis of these disorders have been advanced (e.g., [3]). In addition to clinical features, elevations in serum enzyme levels, and histologic and electromyographic evidence, diagnosis is commonly supported by the presence of characteristic autoantibodies, the nature of which may connote a particular disease subtype or impart prognostic significance [4]. Therapy is normally directed toward immunosuppression, by high-dose corticosteroids alone or in conjunction with steroid-sparing agents such as methotrexate and azathioprine, with addition of further agents (e.g., rituximab, intravenous immunoglobulin (IVIG)) as required for refractory illness. Given the association of malignancy with both PM and DM, particularly the latter [5, 6], a thorough diagnostic work-up for its presence is inevitably prompted by a diagnosis of either condition. In many cases, an occult neoplasm is detected at an early stage and with a higher likelihood of successful treatment. While the lung is a frequent site for primary, myositis-associated malignancy, presentation as a subsolid lesion has not been previously reported. We describe herein a case in which DM was the presenting manifestation of invasive adenocarcinoma of the lung, detected by thin-section computed tomography as an indolent nodule with predominantly ground-glass attenuation.

## Case presentation

**Fig. 1:**
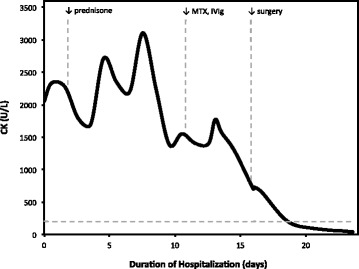
Serum creatine kinase levels are displayed with respect to the duration of hospitalization. The upper limit of normal (200 U/L) is indicated by the dashed horizontal line, while the onset of an intervention is indicated by a dashed vertical line. High-dose prednisone (1 mg/kg/d) was started on hospital day 2; methotrexate (MTX, 7.5 mg/week) and intravenous immunoglobulin (IVIG, 2 g/kg/mo) began on hospital day 11; and right middle lobectomy and mediastinal lymph node dissection took place on hospital day 16. Not shown are escalations of the methotrexate dose to 10 mg/week (hospital day 18) and 15 mg/week (hospital day 25), as well as discharge and commencement of the steroid taper, which coincided on hospital day 29

**Fig. 2:**
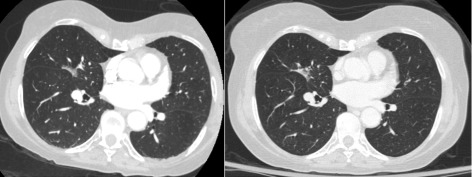
Slices from chest CT scans acquired four years prior to (left) and at the time of presentation (right), demonstrating an indolent, subsolid lesion in the right middle lobe, the solid fraction of which evolved from 15% (left) to 90% (right)

A 72-year-old woman with a history of Graves’ disease treated by radioiodine ablation and no smoking history presented with subacute proximal muscle weakness, accompanied by myalgia and a rash. Dermatologic examination was significant for non-pruritic, macular erythema over the posterior arms; discrete papules on the second and fourth distal interphalangeal joints of the left hand; dusky, reticulated erythema involving the proximal, lateral thighs (evoking the “holster sign” [7]); and linear, erythematous plaques extending inferiorly from the nape. Elevations were noted in the levels of creatine kinase (CK), aldolase, lactate dehydrogenase (LDH), aspartate transaminase (AST), and alanine transaminase (ALT). Suspicion for dermatomyositis prompted initiation of high-dose prednisone (1 mg/kg) on hospital day 2, in spite of which symptoms failed to improve and enzyme levels continued to rise, peaking on hospital day 8 (cf. Fig. 1; peak values were as follows: CK 3108 U/L, aldolase 25.9 U/L, LDH 594 U/L, AST 252 U/L, ALT 344 U/L). Immune serology studies, encompassing general and myositis-specific antibodies, were uniformly negative (ANA, Jo-1, PL-12, PL-7, EJ, OJ, Mi-2, SRP, Ku, U2 snRNP, PM/Scl). Electromyography revealed fibrillations and positive sharp waves, indicative of an irritative myopathic process affecting the proximal muscles. Muscle biopsy demonstrated immune myopathy with perimysial pathology (IMPP). Given the presumptive diagnosis of dermatomyositis, a malignancy work-up was initiated, and computed tomography (CT) of the thorax revealed a 2.1-cm subsolid nodule in the right middle lobe. Relative to a CT scan acquired four years earlier, the nodule was stable in size but had progressed in fractional solidity from 15% (i.e., predominantly ground-glass opacified) to 90% (Fig. 2). PET/CT showed minimal ^18^F-fluorodeoxyglucose (FDG) avidity (standardized uptake value (SUV) 1.3) in the right middle lobe lesion, consistent with its indolent nature, and no evidence of lymph node or metastatic disease. Given the refractoriness of symptoms to high-dose corticosteroids and biopsy evidence of a DM-spectrum process (IMPP), methotrexate (gradually dose-escalated from 7.5 to 15 mg weekly) and IVIG (2 g/kg monthly) were initiated on hospital day 11. Despite the indolence of the pulmonary lesion, the heightened risk of malignancy in the setting of presumed DM prompted thoracoscopic right middle lobectomy and mediastinal lymph node dissection, which the patient underwent on hospital day 16, after spirometry and diffusion capacity testing demonstrated satisfactory pulmonary function. Histopathologic analysis revealed a 2.1-cm, moderately differentiated, invasive adenocarcinoma, arranged in papillary (60%), acinar (30%), micropapillary (5%), and lepidic (5%) proliferative patterns. There was one intraparenchymal node involved by direct extension; level 4R, 7, and 11 lymph nodes were negative for malignancy. Her stage was pT1cN1M0 or IIB (AJCC 7^th^ edition [8]). Molecular studies of the tumor (specifically, short-read sequencing of a target-enriched library of 130 genes that are frequently mutated in solid tumors) disclosed a constitutively activating mutation of EGFR (c.2156G>C; p.G719A) and a mutation of *β*-catenin (CTNNB1) within the GSK3 *β* (GSK3B) phosphorylation region (c.97T>C, p.S33P), with variant allele fractions of 23.5 and 8.6%, respectively. Following surgical excision of the lung tumor, the patient experienced a dramatic improvement in muscle strength and disappearance of the rash. Deltoid and hip-flexor strength increased from 5/10 at the time of surgery to 8/10 within a week. Her enzyme levels continued to decline and were all within the normal range at the time of discharge on hospital day 29. Upon discharge, the corticosteroid dose was tapered from 1 to 0.083 mg/kg/d over a one-month period and the methotrexate discontinued, without relapse of dermatomyositis symptoms; the corticosteroid and IVIG were both discontinued approximately one month thereafter. At the time of writing, the patient continued to show improvements in functional status, and had recently discontinued adjuvant chemotherapy with carboplatin and pemetrexed owing to toxicities.

## Discussion

Although the etiology of DM remains to be elucidated, both innate and adaptive arms of the immune system are thought to play important roles, and autoantibodies — classified as either nonspecific, myositis-associated, or myositis-specific — are found in as many as 80% of patients with PM or DM [9]. Numerous myositis-specific autoantigens have been described, including the signal recognition particle (SRP), the helicase Mi-2, and a battery of aminoacyl tRNA synthetases (e.g., histidyl [Jo-1], threonyl [PL-7], alanyl [PL-12], isoleucyl [OJ], glycyl [EJ]), and autoantibodies directed against each are thought to define distinct myositis syndromes: antisynthetase syndrome, for instance, is strongly associated with interstitial lung disease, while Mi-2 autoantibodies are thought to confer reduced risk for a myositis-associated malignancy [10]. Assaying serum for a set of autoantibodies representing the three aforementioned classes was uniformly negative in the patient described in this report. As a putative autoimmune disease, DM has also been found in association with other autoimmune conditions, including Graves’ disease [11, 12], an association observed in the present case.

The incidence of malignancy is elevated in the setting of DM, an association first recognized in 1916 [13, 14] and confirmed by many retrospective analyses, with standardized incidence ratios (SIRs) typically ranging from 3 to 6 [15–18]. The temporal association of diagnoses of DM and malignancy [16, 18, 19], and the tendency for resolution of DM symptoms upon treatment of the underlying malignancy [6, 20] are consistent with the notion that malignancy-associated DM is a paraneoplastic phenomenon. While the mechanism underlying the association remains ill-defined, tumoral expression of myositis autoantigens has been proposed as a link whereby an immune-response directed against cancer cells leads to inflammatory destruction of antigenically similar muscle tissue [21]. The association of DM with cancer is broad, comprising many histologic types and tissues of origin, the latter of which tends to reflect the anatomic distribution of cancers observed in a population [22]. Accordingly, the lung is a common site of DM-associated cancer among western populations [15]. A review of 24 cases of coincident PM/DM and primary lung cancer spanning the period 1947–2000 found small cell and squamous cell carcinomas to be the most common histologic types [23]. Since then, myriad reports of DM-associated lung cancer have appeared in the literature [24–53]. Combining these reports with those of Fujita et al. [23] gives nearly 50 cases of dermatomyositis-associated, histologically determined lung cancer over the past 70 years, the aggregate analysis of which is presented in Table 1 (many more cases of DM-associated lung cancer have been reported without histologic characterization, e.g., [22]). The most commonly reported histologic type of lung cancer associated with DM is small cell carcinoma (approximately 44%), which is followed by squamous cell carcinoma and adenocarcinoma, each of which account for approximately 17% of reported cases. Given the small, non-systematic sample and the possibility of reporting bias, these frequencies should not be misconstrued as an accurate reflection of the association of lung-cancer histology with dermatomyositis. Among these cases, a history of smoking is common, and in several, including the present one, paraneoplasticity is suggested by the remission of DM shortly after treatment of the associated lung cancer [32, 33, 44, 47].

**Table 1:** Summary of histologic classification of DM-associated lung malignancies reported in the literature (1947–2017) [23–53]

Histology	Cases	Frequency
Small cell carcinoma	21	43.8%
Squamous cell carcinoma	8	16.7%
Adenocarcinoma	8	16.7%
Neuroendocrine	3	6.3%
Undifferentiated	2	4.2%
Other	6	12.5%
Total	48	100.0%

The category “other” comprises mixed histology (e.g., adenosquamous), anaplastic cell carcinoma, alveolar cell carcinoma, giant cell carcinoma, and non-small cell carcinoma not otherwise specified

While previous studies have documented the association between histologic types of lung cancer and DM, little attention has been paid to its association with the molecular basis of malignancy. Genetic analysis of the primary tumor in the present case revealed mutation of the gene encoding EGFR, an event reported to occur in about 10% of cases of non-small cell lung cancer [54]. Point mutations of the codon for G719 account for about 3% of all EGFR mutations in lung cancer; are more common among non-smokers, females, and East Asians [55]; and predict sensitivity to EGFR tyrosine kinase inhibitors [56]. In addition, a mutation was found in the GSK3B phosphorylation region of *β*-catenin (S33P, which leads to constitutive transactivation of T-cell factor and lymphoid enhancer factor target genes). While *β*-catenin has been reported to be rarely subject to mutation in lung cancer [57], recent evidence suggests an important role for Wnt/ *β*-catenin signaling in the progression from lung adenoma to adenocarcinoma [58]. No mutations were found in various other genes implicated in lung adenocarcinoma, including KRAS, ALK, MET, RET, CCND1, TP53, KEAP1, MAP2K1, RIT1, PIK3CA, U2AF1, MDM2, and SETD2 [59]. Whether mutations such as the ones observed bear any relation to paraneoplastic DM remains to be determined.

Finally, the attenuation characteristics of the lesion observed in this case deserve remark inasmuch as an association thereof with paraneoplastic DM is, to our knowledge, without precedent. Ground-glass opacification (GGO) describes an increase in X-ray attenuation which, unlike consolidation, does not obscure underlying vascular and bronchial architecture [60]. Although the finding is generally non-specific, persistence and focality together suggest malignancy, particularly adenocarcinoma or its precursors, the histologic type with which circumscribed subsolid lesions are almost exclusively associated [61, 62]. As a category, subsolid lesions comprise both pure ground-glass-opacified nodules and those with a solid component (part-solid); the GGO component is thought to signify preinvasive growth (atypical adenomatous hyperplasia and adenocarcinoma in situ), and the solid component a nucleus of invasive adenocarcinoma [63]. The consolidation-to-tumor-ratio (CTR), typically defined as the ratio of the maximum linear extents of the solid fraction of the tumor and of the tumor itself, may be used to predict the invasiveness and likelihood of progression of a subsolid lesion [64]. Despite prior claims of association between GGO and EGFR mutation status, a recent meta-analysis found statistically significant association of EGFR mutation status not with pure GGO lesions but with part-solid ones [65]. The majority of subsolid lesions are stable in size; those that grow do so indolently, with reported volume doubling times of 600–900 and 300–500 days for pure GGO and part-solid lesions, respectively [63]. Their typically languorous course explains observations of insignificant FDG uptake [66] — on account of which SUV_*max*_ thresholds must be adjusted for proper interpretation [62] — and moreover demands prolonged (three to five years) surveillance by CT [67, 68]. Indeed, among a cohort of 218 patients with subsolid nodules that had been stable for 3 years, 14 (6.4%) experienced subsequent growth [69], underscoring the need for vigilance. PET/CT, biopsy, and resection should be pursued for nodules having solid components greater than 8 mm in length, expanding consolidation, or other concerning features [68]. Although sublobar resection has been demonstrated to be equally effective to lobectomy for subsolid nodules [67], the higher recurrence rate for part-solid lesions [70] should be taken into consideration when determining surgical management. In the present case, an indolent GGO lesion evolved within four years to EGFR-mutated, non-hypermetabolic, invasive adenocarcinoma, and is, to our knowledge, the first case of a GGO-predominant pulmonary lesion manifesting as DM.

## Conclusions

The case described herein illustrates the importance of maintaining a high index of suspicion for malignancy in the setting of dermatomyositis. The occurrence of a subsolid, indolent, non-hypermetabolic pulmonary lesion, approximately stable in size over a period of four years, in a lifetime nonsmoker may have seemed an unlikely trigger for dermatomyositis, and misapprehension of its significance would have delayed definitive treatment.

## Abbreviations

ALT: Alanine transaminase
AST: Aspartate transaminase
CK: Creatine kinase
CT: Computed tomography
CTR: Consolidation-tumor ratio
DM: Dermatomyositis
EGFR: Epidermal growth factor receptor
FDG: 18F-fluorodeoxyglucose
GGO: Ground-glass opacity
IMPP: Immune myopathy with perimysial pathology
IVIG: Intravenous immunoglobulin
LDH: Lactate dehydrogenase
PET: Positron emission tomography
PM: Polymyositis
SIR: Standardized incidence ratio
SUV: Standardized uptake value
